# Exploring the Functions of 9-Lipoxygenase (*DkLOX3*) in Ultrastructural Changes and Hormonal Stress Response during Persimmon Fruit Storage

**DOI:** 10.3390/ijms18030589

**Published:** 2017-03-15

**Authors:** Kun Meng, Yali Hou, Ye Han, Qiuyan Ban, Yiheng He, Jiangtao Suo, Jingping Rao

**Affiliations:** College of Horticulture, Northwest A&F University, Yangling 712100, China; mengkunxn@163.com (K.M.); houyali1987@163.com (Y.H.); hanyest1989@163.com (Y.H.); banqy1990@126.com (Q.B.); hyh930111@163.com (Y.H.); sjt_9527@163.com (J.S.)

**Keywords:** deletion derivatives, fruit peel, fruit ripening, GUS activity, immunolabelling, promoter

## Abstract

Lipoxygenase (LOX) initiates the hydroperoxidation of polyunsaturated fatty acids and is involved in multiple physiological processes. In this study, investigation of various microscopic techniques showed that the fruit peel cellular microstructure of the two persimmon cultivars differed after 12 days of storage, resulting in fruit weight loss and an increased number and depth of microcracks. Analysis of subcellular localization revealed that greater amounts of DkLOX3-immunolabelled gold particles accumulated in “Fupingjianshi” than in “Ganmaokui” during storage. In addition, the expression of *DkLOX3* was positively up-regulated by abscisic acid (ABA), concomitant with the promotion of ethylene synthesis and loss of firmness, and was suppressed by salicylic acid (SA), concomitant with the maintenance of fruit firmness, inhibition of ethylene production and weight loss. In particular, the expression of *DkLOX3* differed from the ethylene trajectory after methyl jasmonate (MeJA) treatment. Furthermore, we isolated a 1105 bp 5′ flanking region of *DkLOX3* and the activity of promoter deletion derivatives was induced through various hormonal treatments. Promoter sequence *cis*-regulatory elements were analysed, and two conserved hormone-responsive elements were found to be essential for responsiveness to hormonal stress. Overall, these results will provide us with new clues for exploring the functions of *DkLOX3* in fruit ripening and hormonal stress response.

## 1. Introduction

Astringent persimmon (*Diospyros kaki* L.) is an important horticultural commodity with an attractive colour, delicious taste and excellent nutritional properties. International trade and persimmon production are increasing rapidly. However, the fruit softens and decays quickly, negatively affecting its quality and marketability [1]. Moreover, persimmon is classified as a climacteric fruit and ripens quickly, accompanied by maturity-dependent ethylene biosynthesis [2]. Fruit quality is influenced by many factors, which are classified into two major categories: preharvest cultivation and postharvest storage [3]. Fruit ripening is a complex developmental programme that involves many physiological and biochemical modifications, such as membrane deterioration, water loss and chemical changes in the cell wall structure [4,5,6]. Fruit ripening is of particular importance in fruit quality. Therefore, characterization and analysis of ripening-related genes would aid in maintaining postharvest quality and extending the shelf life of fruit [7].

Lipoxygenases (LOX, EC1.13.11.12) are widely distributed in the plant kingdom. The LOX family can be divided into two groups: 9-LOXs and 13-LOXs; they catalyse the oxygenation of polyunsaturated fatty acids (PUFAs) to form a large class of biologically active compounds collectively known as oxylipins, with diverse functions [8,9,10,11]. These functions include regulatory functions in plant developmental processes such as potato tuber growth [12] and Gladiolus corm development [13], resistance to defence [14,15,16], pathogenic fungi [17], high temperature [18] and mechanical wounding [19,20,21,22,23]. In particular, a number of studies have reported the involvement of LOX in fruit ripening, for example, in cucumber [24], melon [25], peach [26], and in the generation of major volatile flavour components as demonstrated in tomato [27,28,29] and kiwifruit [30,31]. The mechanisms involved in fruit ripening have generally been assumed to be associated with membrane deterioration through the hydroperoxidation of PUFAs by LOX, resulting in loss of compartmentalization and cell breakdown [30,32]. Thus, there is a close association between LOXs and fruit quality.

Abundant previous work suggests that ethylene is a primary factor regulating climacteric fruit ripening [4]. Other plant hormones such as abscisic acid (ABA), jasmonic acid (JA), and salicylic acid (SA) are also believed to influence the fruit ripening process [33,34,35,36]. ABA is important for plant growth, development and the response to stress conditions and is able to accelerate the ripening process [37]. Numerous studies have shown that ABA is able to promote ethylene production in many fruits, such as mango [38], strawberry [39] and tomato [40], implying that ABA may function as an upstream regulator of ethylene biosynthesis and responses [41]. JAs, such as methyl jasmonate (MeJA), JA, and other derivatives, make the plant responsive to various biotic and abiotic stresses and are involved in fruit ripening [42,43,44,45]. Jia et al. [46] found that exogenous MeJA mainly regulates the grapefruit ripening process through regulation of fruit colouring, softening, and aroma spreading. SA can be considered a key signalling molecule that delays the postharvest ripening process and extends fruit storability [47], and the ameliorative effects of exogenously applied SA have been observed in plum [48] and apple [49]. Cooperation between the ethylene and JA signalling pathways was confirmed to induce the coordinated expression of a series of ripening-related genes, but SA is generally thought to act antagonistically to ethylene and JA [50]. Therefore, complex regulation occurs among these signalling pathways.

In previous studies, we showed that *DkLOX3*, which belongs to the 9-LOX family and is ethylene/ABA sensitive, could be responsible for ripening and softening in persimmon fruits [51,52]. In addition, we found that *DkLOX3* plays positive roles in the responses to osmotic stress, high salinity and drought via regulating reactive oxygen species accumulation and stress-responsive gene expression [53]. However, there is a lack of available evidence that would allow us to decipher the molecular mechanism of *DkLOX3* in fruit ripening and hormonal stress responses. Hence, in this study, to gain a better understanding of the relationship between *DkLOX3* and fruit ripening, we employed various microscopic techniques to identify qualitative changes in the peel structure of two persimmon cultivars. An important aspect of this work is description of the immunocytolocalization of DkLOX3 in persimmon fruit, as the literature does not provide sufficient data on this issue. Additionally, we isolated the *DkLOX3* promoter and further examined *cis*-regulatory elements to investigate the regulatory mechanisms determining expression under hormonal stress conditions, providing us with new clues for investigating the function of *DkLOX3* gene regulation in hormonal stress responses.

## 2. Results

### 2.1. Physiological Characterization during Persimmon Fruit Storage

**Figure 1:**
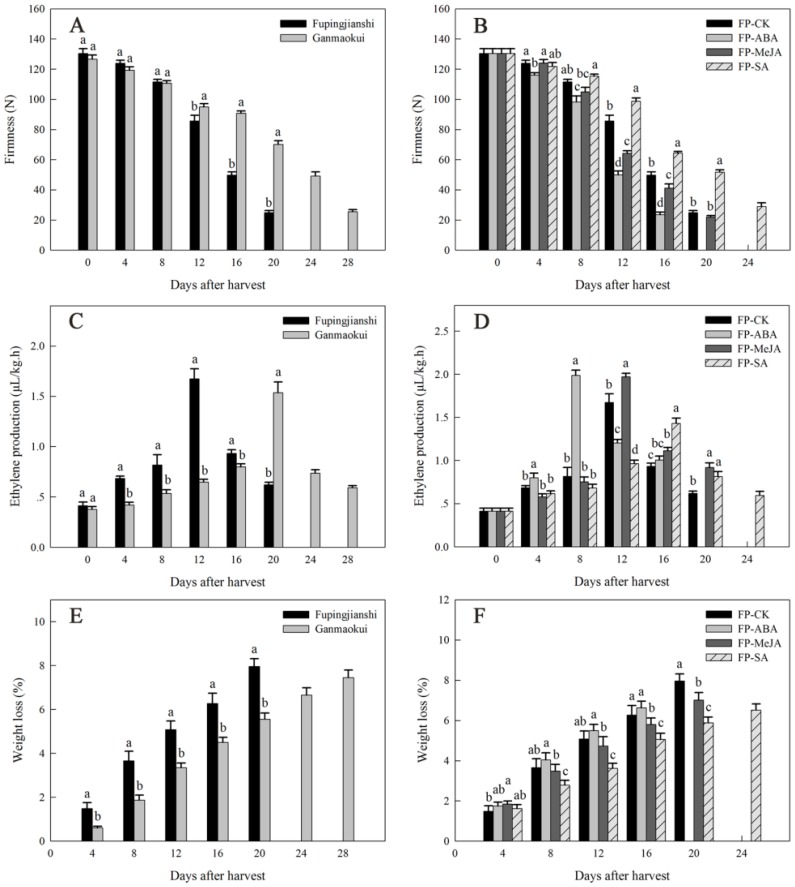
Firmness (**A**,**B**), ethylene production (**C**,**D**) and weight loss (**E**,**F**) in persimmon fruits during storage. (**A**,**C**,**E**) Physiological characterization of two persimmon cultivars, “Fupingjianshi” and “Ganmaokui”. (**B**,**D**,**F**) Physiological characterization of hormone-treated “Fupingjianshi” fruits. FP-ABA, FP-MeJA and FP-SA indicated “Fupingjianshi” fruits treated with ABA (189 µM, 2 min), MeJA (100 µM, 10 min) and SA (100 µM, 10 min), respectively, and stored at 20 ± 1 °C. The fruits were immersed in water and stored at 20 ± 1 °C, served as the FP-CK. Physiological parameters at each time point were calculated from the means of three biological replicates; each replicate included three technical replicates. Vertical bars represent the standard errors of the means. Columns with different letters at each time point indicate significant differences according to the least significant difference (LSD) test (*p* < 0.05).

The firmness during post-harvest ripening has been studied in two persimmon cultivars, “Fupingjianshi” and “Ganmaokui”, which showed similar initial firmness, but differed in the subsequent rate of decline (Figure 1A). “Fupingjianshi” fruits softened rapidly to 24.9 N at the end of the storage period (20 days at 20 °C). “Ganmaokui” fruits exhibited a slower softening trend than “Fupingjianshi”. “Fupingjianshi” fruits have been treated with three hormones, abscisic acid (ABA), methyl jasmonate (MeJA) and salicylic acid (SA) to assay their influence on firmness during storage (stored at 20 °C) (Figure 1B). Compared with FP-CK fruit (“Fupingjianshi” immersed in water), FP-ABA fruit (“Fupingjianshi” treated with ABA) showed a maximum rate of decrease in firmness between days 8 and 12, from 98.3 to 50.0 N (approximately 37%). Additionally, the firmness of FP-MeJA fruit (“Fupingjianshi” treated with MeJA) declined rapidly from 104.9 to 64.2 N (approximately 31.2%) during this period, whereas FP-CK fruit maintained greater firmness. However, FP-SA fruit (“Fupingjianshi” treated with SA) exhibited even greater firmness, being two-fold firmer than FP-CK fruit at 20 days of storage (Figure 1B).

Ethylene production exhibited a typical climacteric pattern in all cultivars and treated fruit during storage (Figure 1C,D). Fruit ethylene production, which was comparable between “Fupingjianshi” and “Ganmaokui” (Figure 1C), was associated with substantial differences in the rate of softening, with maximum ethylene production in these cultivars occurring at day 12 and day 20, respectively. Ethylene production was stimulated by ABA and MeJA, and was suppressed by SA (Figure 1D). The maximum ethylene production observed in FP-ABA fruit (8 days) and FP-MeJA fruit (12 days) was 18.7% and 17.8% higher than in FP-CK fruit (12 days), respectively, whereas the maximum ethylene production in FP-SA fruit (16 days) was only 83.8% of that in FP-CK fruit (12 days), respectively.

Weight loss (associated with the loss of water) was observed throughout storage, and both cultivars and treated fruit showed a gradual loss of weight after harvest (Figure 1E,F). “Fupingjianshi” fruits exhibited a relatively large mean decrease in fresh weight, with 0.40% of weight being lost per day (Figure 1E), whereas “Ganmaokui” fruits showed a less-pronounced increase in weight loss, with only 0.26% of weight being lost per day. The rate of weight loss in FP-CK and hormone-treated fruit was assessed at 20 °C following ambient temperature ripening (Figure 1F). The weight loss was slightly greater in FP-ABA fruit than in FP-CK fruit, although this difference was not statistically significant. In contrast, weight loss in FP-MeJA fruit was slower than in FP-CK, and significant differences were found between the control and treated fruits during the late stages of ripening. Additionally, application of SA significantly delayed weight loss, resulting in a final weight loss of 6.5%; the final weight loss was 81.8% of that in FP-CK fruit.

### 2.2. Structural Analyses of the Persimmon Fruit Peel during Storage

**Figure 2:**
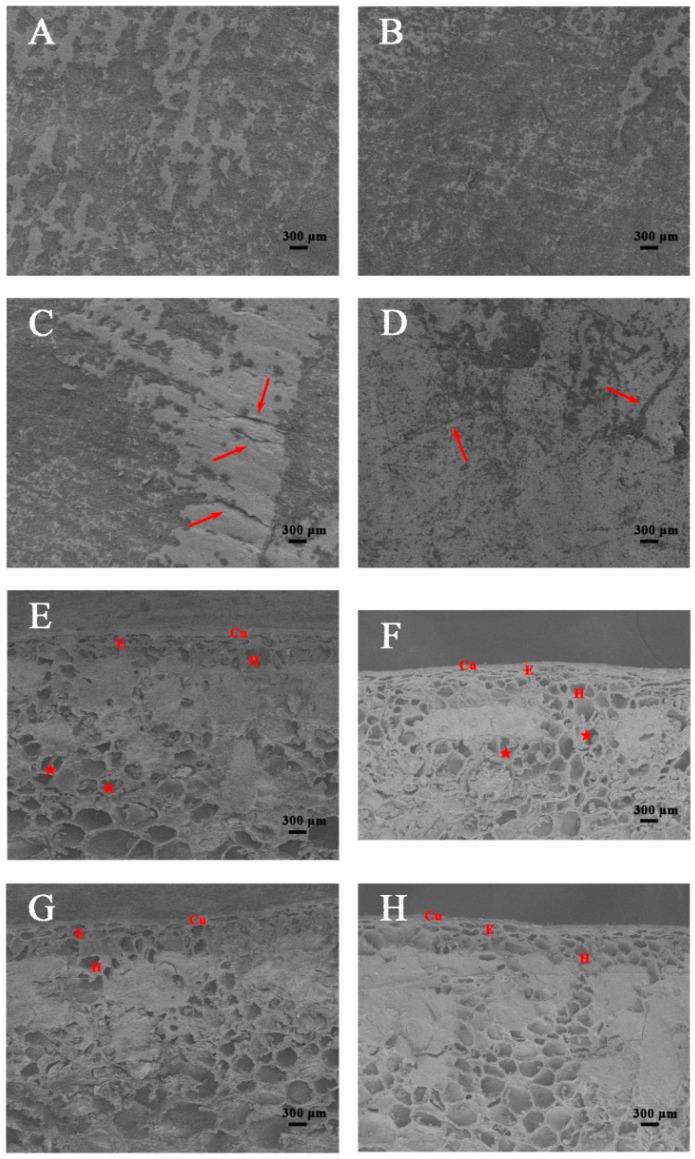
Scanning electron micrographs of fruit peels of two persimmon cultivars during storage. (**A**–**D**) Fruit surface; (**E**–**H**) fragments of the cross-sections through the fruit peel; (**A**,**C**,**E**,**G**) “Fupingjianshi”; (**B**,**D**,**F**,**H**) “Ganmaokui”; (**A**,**B**,**E**,**F**) after harvest; (**C**,**D**,**G**,**H**) after 12 days of storage. Cu cuticle, E epidermis, H hypodermis. Amyloplasts (stars) in hypodermal cells. Arrowheads represent microcracks.

Based on scanning microscopy observations, there was no lenticel visible in the epidermis (Figure 2A,B). The cuticular layer was characterized by the occurrence of microcracks of varying depths and lengths. After 12 days of fruit storage (Figure 2C,D), the number of microcracks on the persimmon surface increased in both cultivars, forming two different patterns. The “Fupingjianshi” fruit had a dry, rough surface covered with fewer microcracks (Figure 2C), although their depth was greater. The “Ganmaokui” fruit exhibited a smooth surface with good continuity, covered with numerous microcracks that did not follow any orientation (Figure 2D), but the depth of the microcracks was shallower than those observed in “Fupingjianshi”, suggesting that the “Ganmaokui” cultivar showed structural characteristics that would extend the storage period. In addition, scanning electron microscopy (SEM) imaging revealed that the structure of the fruit peel in the two persimmon cultivars suffered essential changes during fruit ripening. At harvest, the cells were approximately circular to oval in the fruit peel and contained numerous amyloplasts filled with starch grains (Figure 2E,F), which gradually disappeared during subsequent storage. After the storage period (12 days), the cell morphology became irregular (Figure 2G,H).

**Figure 3:**
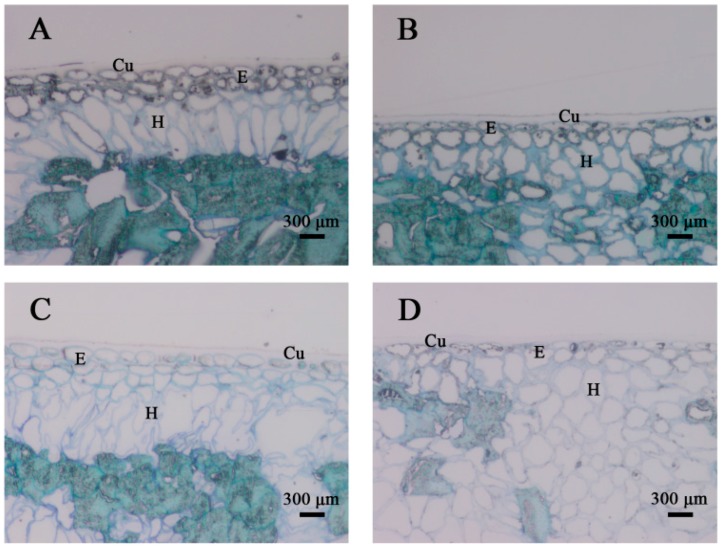
Structural changes occurring in fragments of cross-sections through the fruit peels of two persimmon cultivars during storage. (**A**) “Fupingjianshi”; (**B**) “Ganmaokui”; (**C**) “Fupingjianshi”; (**D**) “Ganmaokui”; (**A**,**B**) after harvest; (**C**,**D**) after 12 days of storage. Cu cuticle, E epidermis, H hypodermis.

To investigate the changes in the cellular structure of the peel, light microscopy was conducted on fruit at harvest and following ripening (Figure 3). The persimmon peel was composed of a single- or double-layered epidermis covered by a cuticle layer and a multi-layered hypodermis. A cuticle layer was often produced not only on the external wall-adhering epidermal cells but also within the internal anticlinal walls in this tissue, in contact with the external environment. The epidermis was composed of small viable cells with a small lumen. Simultaneously, the walls of the epidermal cells were stained densely at harvest (Figure 3A,B). Comparison of the two cultivars showed that the epidermal cells of “Fupingjianshi” presented a slightly greater height than those of “Ganmaokui”, but their width was similar. Following ripening (Figure 3C,D), the lumen of the epidermal cells was increased, and most of the observed cells were turgid. The width of the epidermal cells was similar to that at harvest maturity and their height was slightly greater. In both persimmon cultivars at harvest, the hypodermis consisted of a similar number of layers of collenchyma cells, which appeared closely organized without air spaces between cells (Figure 3A,B). The hypodermal cells of “Fupingjianshi” were characterized by a long oval shape and were approximately arranged longitudinal to the cuticle layer (Figure 3A). In “Ganmaokui”, the hypodermal cells were irregular in shape, with a disordered arrangement (Figure 3B). During the storage period, the cells of the hypodermal layers appeared to be larger, were filled with parietal cytoplasm and exhibited little disruption of their membranes (Figure 3C,D).

### 2.3. Immunocytolocalization of DkLOX3 in Persimmon Fruit during Storage

**Figure 4:**
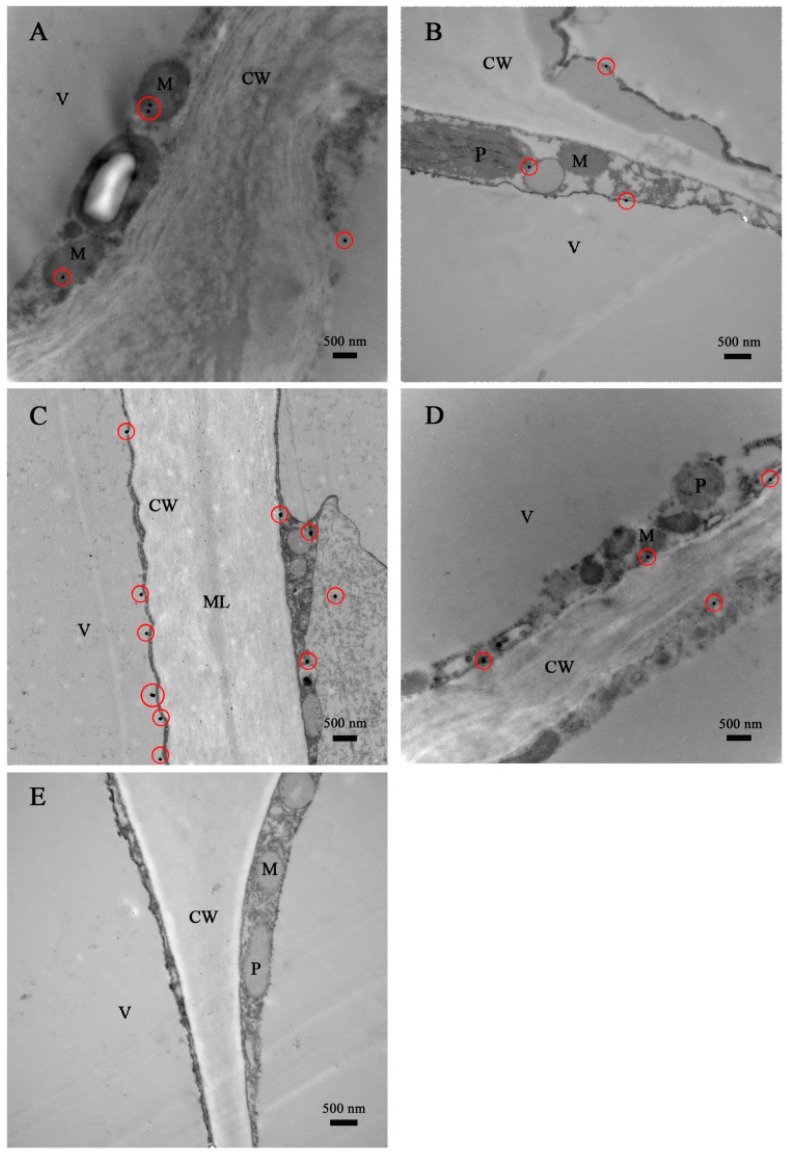
Immunogold electron microscopy localization of DkLOX3 in persimmon fruits from two cultivars during storage. (**A**) “Fupingjianshi”; (**B**) “Ganmaokui”; (**C**) “Fupingjianshi”; (**D**) “Ganmaokui”; (**E**) control; (**A**,**B**) after harvest; (**C**,**D**) after 12 days of storage. CW cell wall, ML middle lamella, M mitochondrion, V vacuole, P plastid. Gold particles are encircled in red. (**A**) 20,000×; (**B**) 25,000×; (**C**) 20,000×; (**D**) 20,000×; (**E**) 25,000×.

To demonstrate the relationship between DkLOX3 and ultrastructural changes, the subcellular localization of DkLOX3 in persimmon fruit was determined using the immunogold electron-microscopy technique (Figure 4). DkLOX3, visualized using gold particles, was primarily present in the cytoplasm, plastids and mitochondria. At harvest maturity, transmission electron microscopy (TEM) images indicated that the cell wall structure was complete, with a high-density middle lamella, appearing as a bright–dark–bright partition structure (Figure 4C). The mature cells were characterized by one large vacuole and dense cytoplasm containing numerous organelles that showed structural integrity, with a plasmalemma. A small number of gold particles immunolabelled with an antibody against DkLOX3 were found in the two persimmon cultivars at harvest (Figure 4A,B). After the storage period, the cell wall was notably deteriorated, displaying loss of electron density, and the structure of the plasmalemma was blurry (Figure 4C,D). Abundant gold particles accumulated in the cell plasmalemma of “Fupingjianshi” (Figure 4C), whereas few immunolabelled gold particles were observed in “Ganmaokui”, similar to what was observed at harvest maturity (Figure 4D). Furthermore, no gold particles were found when the polyclonal antibody against DkLOX3 was omitted during immunolabelling (Figure 4E), suggesting that the immunogold electron-microscopy localization observed in the experiment was both specific and reliable.

### 2.4. Expression of DkLOX3 during Persimmon Fruit Storage

**Figure 5:**
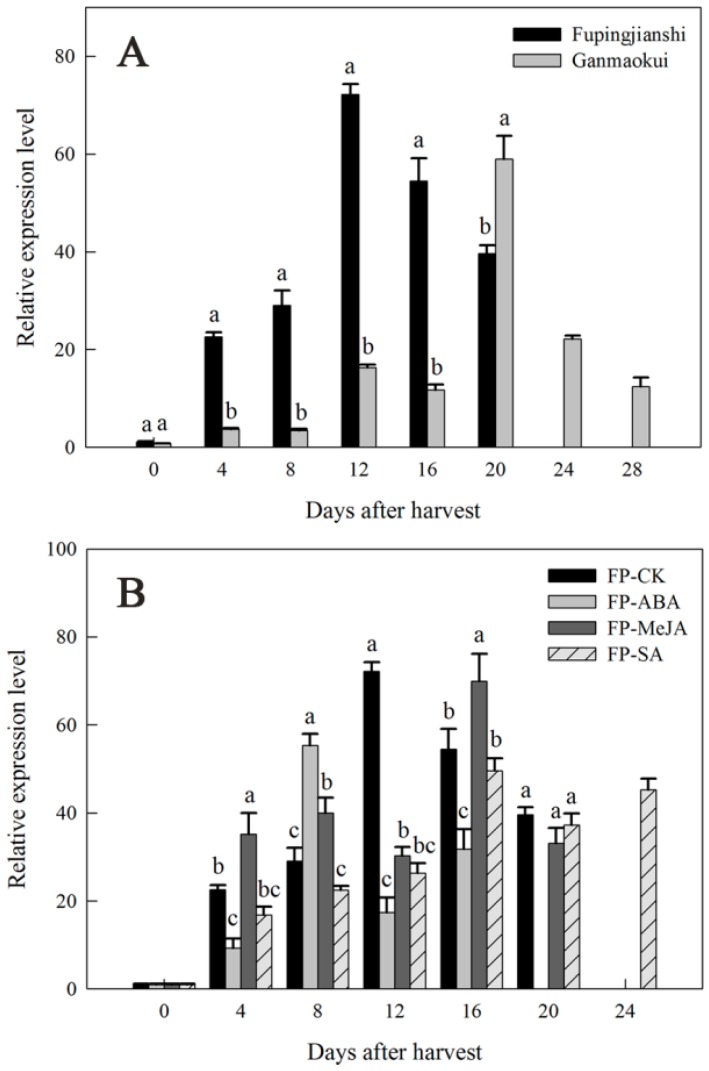
Expression pattern of *DkLOX3* in persimmon fruits during storage. (**A**) Expression pattern of *DkLOX3* in two persimmon cultivars; (**B**) expression pattern of *DkLOX3* in hormone-treated “Fupingjianshi” fruits. FP-ABA, FP-MeJA and FP-SA indicated “Fupingjianshi” fruits treated with ABA (189 µM, 2 min), MeJA (100 µM, 10 min) and SA (100 µM, 10 min), respectively, and stored at 20 ± 1 °C. The fruits were immersed in water and stored at 20 ± 1 °C, served as the FP-CK. The values for each time point represent the average from three PCR runs for three biological replicates. Vertical bars represent standard errors of means. Columns with different letters at each time point indicate significant differences according to the LSD test (*p* < 0.05).

The physiological roles of the persimmon *DkLOX3* genes were investigated in the two cultivars and under different hormonal treatments by monitoring changes in transcript levels using quantitative real-time polymerase chain reaction (qPT-PCR) (Figure 5). The pattern of the increase in transcript abundance of *DkLOX3* appeared to parallel the ethylene trajectory, with maximum levels occurring during or within several days of peak ethylene production (Figure 1C,D). The expression of *DkLOX3* in “Fupingjianshi” increased sharply and reached a peak on the same day (12 days) as ethylene production, then decreased towards the late stages of ripening (Figure 5A). The expression level in “Ganmaokui” also increased several-fold during late ripening, reaching a maximum at 20 days after harvest that was considerably lower than in “Fupingjianshi”. *DkLOX3* expression was stimulated strongly in FP-ABA fruit and was evidently suppressed in FP-SA fruit, which maintained lower expression levels during ripening (Figure 5B). Additionally, in FP-ABA fruit, the expression of *DkLOX3* peaked at 8 days, 4 days ahead of that in FP-CK fruit (12 days), whereas peak expression of *DkLOX3* in FP-SA was delayed by 4 days, compared with that of FP-CK. Interestingly, after the application of MeJA, *DkLOX3* peak expression occurred 4 days later and was lower than in FP-CK but increased to a higher level than in FP-CK during the early stages of ripening (Figure 5B). Therefore, the *DkLOX3* expression pattern in FP-MeJA was obviously different from the ethylene trajectory.

### 2.5. Isolation and Sequence Analysis of the DkLOX3 Promoter

**Figure 6:**
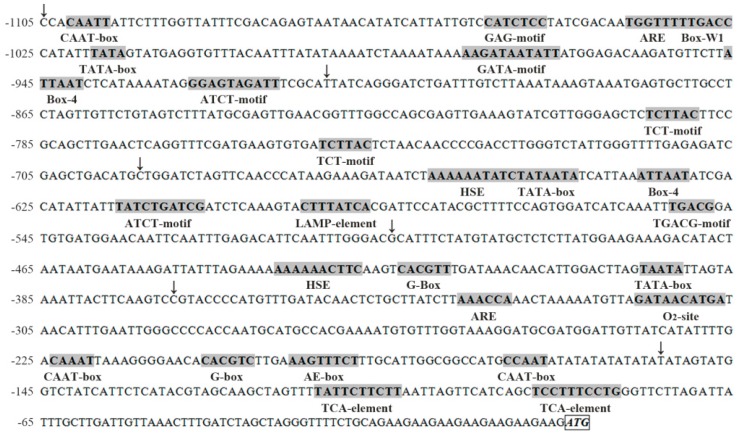
Nucleotide sequence of the *DkLOX3* promoter. The *DkLOX3* translational start codon ATG (bold, italic and boxed) numbered as ±1. Putative regulatory elements are shown in bold and shaded in grey; names are given below the elements. Arrowheads represent the starting point of 5′-deleted derivatives.

To explore the regulation of *DkLOX3*, the 1105 bp 5′ flanking region designated *pDkLOX3* (GenBank accession number KX779272) was isolated from “Fupingjianshi” via genome walking and was analysed for putative *cis*-regulatory elements using the PlantCARE database [54]. The *pDkLOX3* promoter sequence and putative plant regulatory elements are shown in Figure 6. Sequence analysis revealed that the *DkLOX3* promoter region contained various putative plant regulatory elements. Furthermore, a detailed analysis of the *cis*-regulatory elements within the promoter enabled us to classify them into four functional groups: abiotic stress-, biotic stress-, hormone response- and light response-related elements. Heat stress-responsive elements (HSEs) [55] are an important type of abiotic stress-responsive element. The biotic stress-responsive elements consisted of anaerobic-responsive elements (AREs) [56], an element involved in the regulation of zein metabolism (O_2_ site) [57], and a fungal elicitor responsive element (Box-W1) [58]. The hormone-responsive elements included a MeJA-responsive element (TGACG motif) [59] and two salicylic acid (SA)-responsive elements (TCA elements) [60]. The light-responsive elements consisted of a G-box [61] and other typical elements, including an AE box, ATCT motifs, a GAG motif, and a LAMP element. These putative *cis*-regulatory elements indicated that *DkLOX3* might be partially involved in the response to environmental changes and hormone signalling.

### 2.6. Characterization of DkLOX3 Promoter Activity in Tobacco Leaves

**Figure 7:**
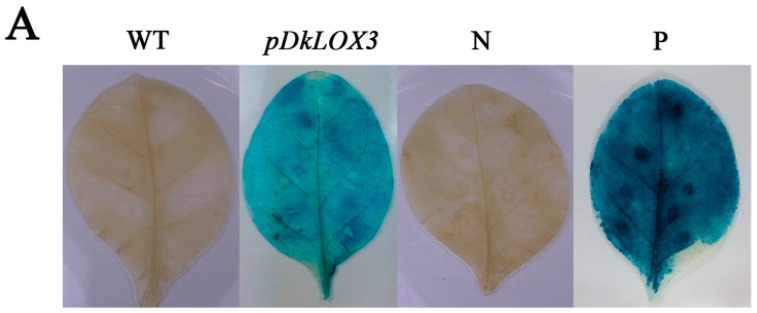

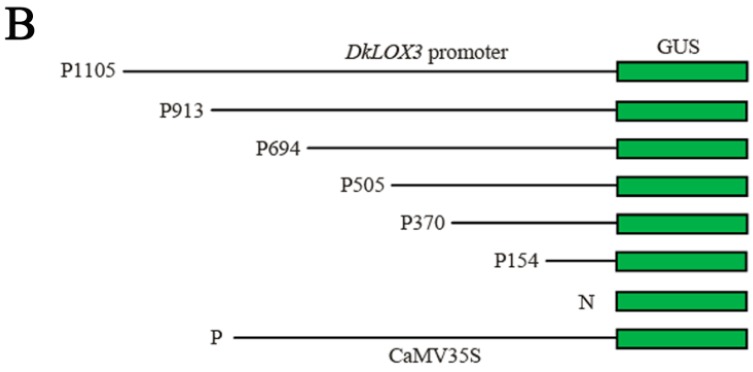
Histochemical staining (**A**) of transiently transformed tobacco leaves and schematic diagram of vector constructs (**B**) for the *DkLOX3* promoter. WT wild type; N negative control; P positive control.

To test the activity of *pDkLOX3*, the promoter-GUS fusion construct pDkLOX3:GUS was analysed in an *Agrobacterium*-mediated transient expression system. A CaMV35S:GUS (pBI121-35S-GUS) construct was used as the positive control, and a promoterless construct (pBI121-GUS) served as the negative control. No GUS activity was observed in wild type (WT). A histochemical assay verified that the *DkLOX3* promoter was able to drive the expression of the GUS reporter gene (Figure 7A), even though the promoter activity of *DkLOX3* was much lower than that of the positive control.

### 2.7. Responsiveness of DkLOX3 Promoters to Hormonal Stress

**Figure 8:**
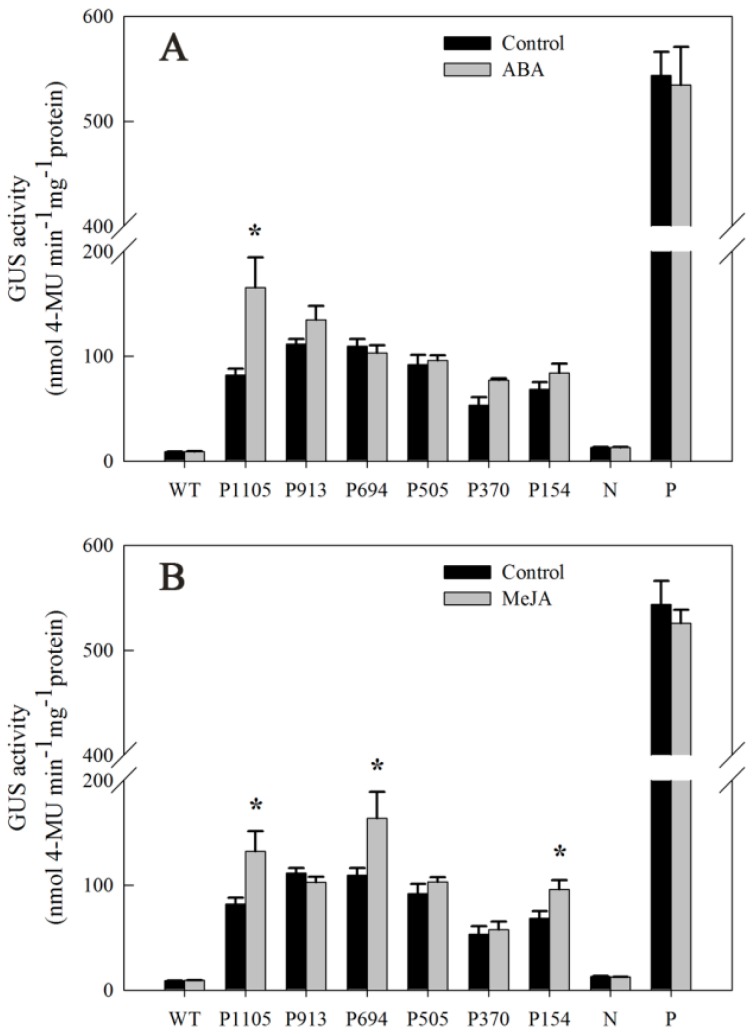

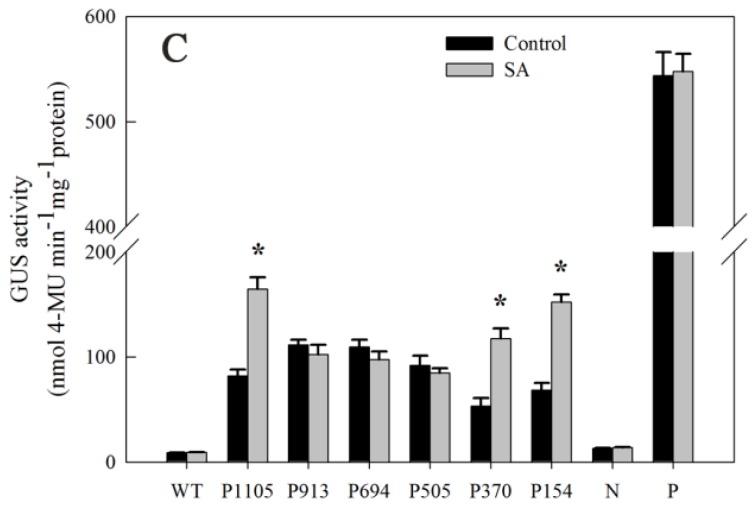
Analysis of GUS activity from promoters in response to hormonal treatments in transiently transformed tobacco leaves. (**A**) Sprayed with 189 µM ABA; (**B**) Sprayed with 100 µM MeJA; (**C**) Sprayed with 100 µM SA. Mean activity was averaged from three independent experiments. Vertical bars represent the standard errors of means. Stars (*) represent significant differences according to the LSD test (*p* < 0.05).

To elucidate whether the differential gene expression patterns of *DkLOX3* are correlated with the regulation of elements in its promoter, we prepared a series of *pDkLOX3* deletions and fused them to the promoterless GUS reporter gene (Figure 7B). Each construct was introduced into tobacco leaves, and its activity under various hormonal stress conditions was investigated (Figure 8). Compared with the control, the GUS activity of pDkLOX3:GUS was substantially increased by ABA, MeJA and SA by approximately 2.02-fold, 1.62-fold and 2.0-fold, respectively. In detail, significant ABA-inducible promoter activity was detected only for the P1105 construct (Figure 8A), while evident MeJA-inducible promoter activity was detected in tobacco leaves harbouring the P1105, P694, and P154 constructs (Figure 8B). Additionally, significant SA-inducible promoter activity was detected for the P1105, P370, and P154 constructs (Figure 8C). In addition, in all of the treatments, wild-type leaves and those transformed with the positive construct showed no obvious inducible GUS activity compared with that of the controls. These results indicated that the *DkLOX3* promoter was induced by hormonal stress.

## 3. Discussion

LOXs, encoded by a large multigene family with different individual functions, play an important role in fruit ripening [29,30]. However, the existing evidence does not provide sufficient data regarding the molecular mechanism of 9-LOX gene regulation in fruit ripening. In this study, we explored the roles of the 9-LOX gene *DkLOX3* in ultrastructural changes and hormonal stress response in persimmon fruit ripening.

### 3.1. DkLOX3 May Have a Positive Role in Ultrastructural Changes during Fruit Ripening

For successful commercialization, in addition to favourable climatic and cultivation conditions, proper timing of the harvest and proper storage conditions are vital for ensuring high fruit quality. Also important is the genetic background, which determines the structural changes that take place in the fruit peel, influencing texture, flavour, appearance, water loss and nutritional properties during fruit ripening [62]. In the present study, the fact that the initial (0 day) parameters, including ethylene production and firmness, were comparable in the two cultivars provides evidence that “Fupingjianshi” and “Ganmaokui” were at similar maturity at harvest (Figure 1A,C). During subsequent storage, the ripening patterns of the cultivars diverged. “Fupingjianshi” exhibited poorer and shorter storability, losing firmness faster than “Ganmaokui” (Figure 1A). In addition to showing differences in ethylene sensitivity (Figure 1C), the cellular microstructures of the two cultivars were quite different in the fruit peel (Figure 2 and Figure 3), and the significant differences between the cultivars in terms of the structural changes observed in various tissues (the cuticle, the epidermis and the hypodermis) during storage can be responsible for different ripening rates.

The cuticle, a thin extracellular polymeric membrane with a layered structure, plays the most important protective role against adverse environmental conditions [63] and is a key factor determining important traits related to fruit postharvest quality, such as water loss [64]. Konaraka [65] reported that additional cuticle deposition was detected on the internal wall of the epidermis after the storage period, thus increasing the thickness of the cuticle layer in two varieties of apples. In our light microscopy measurements of the persimmon fruit peel (Figure 3), the cuticle thickness was not obviously different at harvest and during storage. In the present study, 12 days of storage resulted in fruit weight loss (Figure 1E) and increased the numbers and depths of microcracks in both persimmon varieties (Figure 2C,D), which did not exhibit any evident cuticle microcracks at harvest. The microcrack depth was greater in “Fupingjianshi” fruit than in “Ganmaokui” fruit, accompanied by more rapid loss of firmness and weight (Figure 1A,E). Konaraka [65] and Curry [66] reported similar observations. With the absence of lenticels in persimmon fruit peel (Figure 2A,B), most water transpires through the cuticle and its microcracks, leading to decreasing fruit weight and firmness. This phenomenon has been discussed by Veraverbake et al. [67], who attributed the major role in water transpiration to inner epidermal cells. In addition to the internal characteristics of the cuticle and its transport properties, the amount of transpired water also depends on conditions prevailing in the storehouse (temperature, O_2_, CO_2_ and humidity) [68]. The literature suggests that microcracks enhance cuticular transpiration [69], forming a direct channel between internal cells and external environments that facilitates gas exchange. During persimmon fruit storage, exogenous O_2_ is transported through microcracks into internal tissues. This process could accelerate the oxygenation of PUFAs associated with 9-LOX DkLOX3, resulting in membrane deterioration and loss of compartmentalization. However, many more experiments are needed to directly elucidate this hypothesis.

In our immunogold localization experiments, DkLOX3 was primarily present in the membranes surrounding the cytoplasm, plastids and mitochondria (Figure 4). Relevant analyses of subcellular localization have shown that LOX localizes to a variety of subcellular structures, such as the chloroplasts [70], liposomes and vacuoles [71], associated with diverse functions. In addition, many gold particles accumulated in the cell plasmalemma of “Fupingjianshi” (Figure 4C), whereas few immunolabelled gold particles were observed in “Ganmaokui” (Figure 4D), and “Fupingjianshi” ripened much faster than “Ganmaokui”. This result was consistent with the expression level of *DkLOX3*, as *DkLOX3* expression in “Fupingjianshi” was stimulated more rapidly than in “Ganmaokui” (Figure 5A). These results suggested that DkLOX3 may play a positive role in the ultrastructural changes promoting persimmon fruit ripening. The relationship between subcellular localization and physiological function is a hot topic in enzymology research. To our knowledge, this was the first study to successfully localize LOX in persimmon fruit. 

### 3.2. DkLOX3 May Play an Important Role in Hormonal Stress Response during Fruit Ripening

Fruit ripening is a complex developmental programme regulated by various genetic factors and biochemical pathways [6]. Although ethylene plays a primary role in regulating climacteric fruit ripening, accumulating evidence has revealed that fruit ripening is not simply modulated by individual hormones but is regulated by many different phytohormones through a complicated network of feedback and crosstalk [72,73,74]. As promoters play an important role in initiating gene transcription and regulate gene expression temporally and spatially, good knowledge of the pattern of promoter activity is necessary in studying gene function [75]. The combination of exogenous treatments and promoter analysis employed in the present study may provide an integrated representation of the roles of *DkLOX3* in the hormone response in the regulation of fruit ripening.

In agreement with previous studies, our results showed that application of ABA accelerated persimmon fruit ripening, concomitant with promoting ethylene synthesis and loss of firmness (Figure 1B,D) [38,40,76]. Additionally, exogenous ABA was associated with enhanced transcriptional levels of *DkLOX3* during fruit ripening (Figure 5B). Some evidence has suggested that ethylene and ABA play fairly important roles in the control of fruit ripening [38,40,73,77]. In tomato and peach fruits, ABA promotes ripening by inducing ethylene biosynthesis through the up-regulation of ethylene biosynthesis genes [76]. So we deduced that exogenous ABA may indirectly up-regulate *DkLOX3* expression via synergistic effects with ethylene. In addition, our data from the ABA experiment also showed that the entire P1105 construct was significantly activated when ABA was applied to tobacco leaves (Figure 8A). The result suggested that the *DkLOX3* promoter was an inducible promoter. However, sequence analysis revealed that there were no ABA-responsive elements (ABREs) in the *DkLOX3* promoter (Figure 6). In general, some functional genes respond to abiotic stress using ABA-dependent or ABA-independent pathways via ABREs [78]. Even if inducible by ABA, the functional genes with no ABREs are regulated by recognition motifs of a transcription factor. Therefore, we speculated that the activity of the *DkLOX3* promoter might be regulated by the interactions between transcription factors and *cis*-regulatory elements in tobacco leaves. These results imply that *DkLOX3* was indirectly up-regulated by exogenous ABA, perhaps via promoter regulation, thereby promoting fruit ripening. However, further studies are needed to verify this speculation.

In the present study, we found that MeJA plays a positive role in persimmon fruit ripening, accelerating ethylene synthesis and the loss of firmness (Figure 1B,D). Similar results have been reported in peach [26] and grape [46]. In peach, the peak of ethylene release occurs earlier after MeJA treatment. Jia et al. [46] revealed that JA plays an important role in grapefruit softening by increasing the transcription levels of several ripening-related genes. In the present study, treatment with MeJA resulted in an obvious increase in *DkLOX3* expression during persimmon fruit ripening (Figure 5B). In addition, the GUS activity of the P1105, P694, and P154 constructs was strongly induced (Figure 8B). We noted the presence of a TGACG motif in the promoter sequence between −694 and −505 (Figure 6), which is a MeJA-responsive *cis*-regulatory element [59]. Therefore, these combined results demonstrate that exogenous MeJA regulates the expression of *DkLOX3* via the TGACG motif.

Postharvest treatment with SA maintained fruit firmness, inhibited ethylene production and reduced weight loss, which appeared to effectively delay persimmon fruit ripening (Figure 1B,D,F). These results are congruent with early studies in peach [79], plum [48] and strawberry [80]. Exogenous application of SA has been found to act antagonistically to ethylene [50], either repressing the expression of the ACS and ACO genes or resulting in reduced activity of related enzymes, thus delaying ripening in kiwifruit [33] and tomato [81]. In addition, Giménez et al. [82] demonstrated that MeSA application is an effective tool for improving sweet cherry fruit quality characteristics during storage, enhancing bioactive compound concentrations and antioxidant activity. In the present study, treatment with SA resulted in obvious down-regulation of *DkLOX3* expression (Figure 5B). Additionally, the GUS activity of the P1105, P370, and P154 constructs was strongly induced by SA treatment, whereas the GUS activity of the P913, P694, and P505 constructs slightly declined (Figure 8C). We also identified two TCA elements in the P154 construct (Figure 6). We deduced that the TCA element plays an important role in the *DkLOX3* promoter. These results demonstrate that this element may assist in regulating the expression of *DkLOX3* when SA acts as a signalling molecule.

## 4. Materials and Methods

### 4.1. Plant Materials and Treatments

Two astringent persimmon fruit cultivars, “Fupingjianshi” and “Ganmaokui”, with different postharvest ripening rates [83] were obtained at the onset of ripening (70%–80% surface yellow coloration) from commercial orchards in Fuping County and Xiaoyi County, in Shaanxi Province of China, respectively, and then transported within hours to the postharvest facilities at Northwest A&F University. Uniform fruits without mechanical damage that were free of visible defects or decay were chosen for the following experiments.

The selected “Fupingjianshi” fruits were divided randomly into four experimental groups, with 300 fruits in each group. The first group, which was immersed in water, served as the control. The second and third groups were immersed in 100 µM MeJA (Sigma-Aldrich, St. Louis, MO, USA) or 100 µM SA (Sigma-Aldrich) for 10 min, respectively. The fourth group was immersed in 189 µM ABA (Sigma-Aldrich) for 2 min. After treatment, the fruits of each group were stored at ambient temperature (20 ± 1 °C). Additionally, the firmer persimmon fruit cultivar “Ganmaokui”, harvested at similar maturity without receiving any treatment, was stored at ambient temperature (20 ± 1 °C). All of the treated and non-treated fruits were randomly divided into three subgroups. Samples from each subgroup were collected at 4-day intervals for the determination of firmness, ethylene production and weight loss, starting on the day of harvest and continuing through softening of the flesh. At each sampling, pooled flesh tissues (without the skin and core) were cut into small pieces, immediately frozen in liquid nitrogen, and stored at −80 °C until use.

Tobacco plants (*Nicotiana tabacum* cv. NC89) were cultured in a controlled-environment growth chamber under a 16/8 h photoperiod, with 65% relative humidity and a 25/20 °C (day/night) temperature cycle. Six-week-old plants were used for *Agrobacterium*-mediated transient assays. In one set of treatments, at 48 h after infiltration, tobacco plants were sprayed with the same hormones (100 µM MeJA, 100 µM SA or 50 mg·L^−1^ ABA) applied to the persimmon fruits or with sterile water (control). After treatment, all tobacco plants were maintained in a chamber under a 16/8 h photoperiod, with humid conditions, at 28 °C for 24 h. Leaf samples from all treatments were subsequently collected for the assessment of the GUS activity of the promoters. All experiments were repeated at least three times.

### 4.2. Fruit Firmness, Ethylene Production and Weight Loss

Fruit firmness was measured with the pericarp removed at two equidistant points on the equatorial axis of 10 fruits. Firmness was determined with a pressure tester (Model FT327; Effegi, Milan, Italy) equipped with a 5-mm diameter flat probe. Firmness is expressed as N.

To measure ethylene production, six fruits from each cultivar and treatment subgroup were enclosed and sealed in a 3.6-L vessel for 1 h at storage temperature. Then, a gas sample (1 mL) was withdrawn from the headspace using a syringe. Ethylene production was determined by injecting a gas sample into a flame ionization detection GC-14A gas chromatograph (Shimadzu, Kyoto, Japan), as described by Meng et al. [52]. Ethylene production is expressed as µL·kg^−1^·h^−1^.

Six fruits from each cultivar and treatment subgroup were separately marked before storage. Weight loss during the storage period was recorded on a digital balance starting at the time point of harvesting; thereafter, the same fruits were consistently weighed on each sampling date. Weight loss was expressed as a percentage and was calculated using the following formula [84]: weight loss % = (fruit initial weight − fruit weight at each sampling date) × 100/fruit initial weight.

### 4.3. Structural Analyses

At harvest and after 12 days of storage, cross-sections perpendicular to the fruit equatorial axis were hand-cut through the fresh peels of five “Fupingjianshi” and “Ganmaokui” fruits matched for size and ripeness using a razor blade. Pieces of persimmon fruit tissue of 5 mm^3^ were immersed in a fixative solution containing 2.5% (*v*/*v*) glutaraldehyde and 2% (*w*/*v*) potassium antimonate in 0.1 mM phosphate buffer (pH 7.2) for 24 h at 4 °C. Subsequently, the samples were washed with phosphate-buffered saline (PBS) buffer (pH 7.2) three times, post-fixed in 1% (*w*/*v*) osmium tetroxide for 3 h at 4 °C, dehydrated through an ethanol series (30%–100%), and dried at the critical point in liquid CO_2_. After sputter coating with a 10 nm-thick gold-palladium alloy, the samples were examined with an S-4800 scanning electron microscope (Hitachi, Japan) using a 2 kV accelerating voltage [85].

For light microscopy (LM) observations, multiple fragments (2 mm^3^) with peels from multiple persimmon fruits (*n* ≥ 5) were embedded in Epon812 resin (SPI Supplies Division of Structure Probe, Inc., West Chester, PA, USA). Semi-thin (1.0 µm) transverse sections (perpendicular to the fruit pericarp) were cut on a Leica Ultracut R ultramicrotome (Leica, Wetzlar, Germany) with a diamond knife and stained with 1% toluidine blue in borate buffer (pH 4.4). The samples were observed under an Olympus BX43 microscope (Olympus, Tokyo, Japan).

### 4.4. Immunolabelling for Transmission Electron Microscopy (TEM)

The procedure for fruit tissue preparation was adapted from above, with small pieces of persimmon (2 mm^3^) being cut, fixed overnight in a fixation solution and dehydrated through a graded ethanol–water series. The samples were subsequently embedded in Epon812 resin and polymerized for 48 h at 65 °C. Ultra-thin sections (approximately 75 nm thick) were cut with a Leica Ultracut R ultramicrotome using a diamond knife and collected on copper Formvar-coated grids (230 mesh). To examine DkLOX3 localization, the immunostaining procedure of Sutherland et al. [86] was adapted. The rabbit polyclonal DkLOX3-Ab antibody was prepared by GenScript (Nanjing, China). Sections were incubated in an appropriate dilution of DkLOX3-Ab in PBS buffer for 1.5 h, then washed three times for 10 min each. Next, the grids were incubated with goat anti-rabbit IgG conjugated to 10 nm-diameter colloidal gold particles for 1 h at room temperature. After a thorough wash in distilled water, all sections were double stained with a 5% (*w*/*v*) uranyl acetate (20 min) and 2% (*w*/*v*) lead citrate solution (5 min). Controls without primary or secondary antibodies were also performed. The sections were then analysed with a transmission electron microscope (H-7650, Hitachi, Japan) at an 80 kV accelerating voltage.

### 4.5. Quantitative Real-Time PCR Analysis

Total RNA was extracted from frozen persimmon tissues according to the hot borate method [87], and its concentration and integrity were checked. Synthesis of first-strand cDNA was performed using 1.0 µg of RNA as a template and the PrimeScript^TM^ RT Reagent Kit with gDNA Eraser (TaKaRa, Dalian, China), following the manufacturer’s instructions. The expression of persimmon *Actin* (GenBank ID AB219402), as a housekeeping gene, was used as an endogenous reference to minimize variation in cDNA template levels. The specificity of the *DkLOX3* and *Actin* primers was determined using melting curves and through PCR product resequencing. The primer sequences used for quantitative real-time PCR (qRT-PCR) are listed in Table 1.

qRT-PCR was performed using an iCycler iQ5 (Bio-Rad, Hercules, CA, USA) for gene expression analysis. The PCR mixture (20 µL total volume) comprised 1.0 µL of diluted cDNA (300 ng/µL), 7.4 µL of ddH_2_O, 0.8 µL of the sense primer and antisense primers (10 µmol·L^−1^), and 10 µL of 2× SYBR Premix Ex Taq II (TaKaRa, Dalian, China). No-template controls for each primer pair were included in each run. The PCR program was initiated at 95 °C for 3 min, followed by 40 three-step cycles of template denaturation at 95 °C for 10 s, primer annealing at 55 °C for 30 s, and extension at 72 °C for 20 s. Relative target gene expression levels were calculated according to the 2^−ΔΔ^*^C^*^t^ method [88] using iQ5 2.0 (Bio-Rad) standard optical system analysis software. The expression level at the time point of harvesting in early-ripening “Fupingjianshi” fruit was expressed as the calibrator, which was set to 1. RNA isolation and cDNA synthesis were performed at least three different times as biological replicates for qRT-PCR.

### 4.6. Cloning of the 5′ Flanking Region of DkLOX3

Frozen persimmon tissue was ground in an extraction buffer consisting of 100 mM Tris-Cl (pH 8.0), 2% (*v*/*v*) hexadecyltrimethylammonium bromide (CTAB), 1.4 M NaCl, 50 mM ethylene diamine tetraacetic acid (EDTA) and 2% (*v*/*v*) β-mercaptoethanol (β-ME). Genomic DNA was extracted using phenol/chloroform, precipitated with ethanol, and dissolved in TE buffer (10 mM Tris-HCl, 1 mM EDTA, pH 8.0). The concentration, quality and integrity of the obtained DNA were analysed using a NanoDrop^®^ND-1000 spectrophotometer (Nanodrop Technologies, Wilmington, DE, USA) and through agarose gel (0.8%) electrophoresis. A fragment of the 5′ upstream flanking region including the translation start codon of *DkLOX3* was isolated from the “Fupingjianshi” genomic DNA with the Genome Walking Kit (TaKaRa, Dalian, China), following the manufacturer’s instructions. For nested PCR, the *DkLOX3* gene-specific primers pLOX3-SP1, pLOX3-SP2 and pLOX3-SP3 were used (Table 1). The amplification product was purified, cloned into the pMD18-T vector (TaKaRa, Dalian, China), and sequenced by GenScript (Nanjing, China). The promoter sequence was analysed using the PlantCARE (http://bioinformatics.psb.ugent.be/webtools/plantcare/html/) database [54].

### 4.7. Construction of the Promoter-GUS Fusion

Two expression vectors, pBI121-GUS and pBI121-35S-GUS, were constructed for transient expression assays. The 5′ flanking region of *DkLOX3* was generated through PCR amplification using the gene-specific primers DkLOX3-PF/DkLOX3-PR. In addition, a series of nested 5′ deletions of *pDkLOX3* fragments were generated via PCR amplification. Five forward primers (DkLOX3-PF1 through DkLOX3-PF5, Table 1) were designed to correspond to the −913, −694, −505, −370, and −154 sequences of the *DkLOX3* promoter. Together with the gene-specific primer DkLOX3-PF, all forward primers were extended with a *Hind*III restriction enzyme site (underlined sequences), while an *Xba*I site (underlined) was added to the 5′ end of the reverse primer DkLOX3-PR. To construct the pDkLOX3:GUS plasmid, each promoter fragment was double digested with *Hind*III/*Xba*I and ligated into the *Hind*III/*Xba*I site of the vector pBI121-35S-GUS. The recombinant was transformed into *E. coli* DH5α and cultivated on an Luria-Bertani (LB) kanamycin plate. The purified recombinant plasmid was identified through restriction enzyme analysis and sequencing (GenScript, Nanjing, China). Subsequently, the verified fusion constructs were introduced into *Agrobacterium tumefaciens* strain EHA105 via the freeze-thaw method. A schematic representation of the promoter deletions is shown in Figure 7B.

### 4.8. Agrobacterium-Mediated Transient Expression Assays in Tobacco Plants

*Agrobacterium*-mediated transient expression assays were performed as previously described [89]. The *A. tumefaciens* strain EHA105, containing the promoter constructs, was expanded and cultivated in LB liquid medium supplemented with rifampicin (60 mg·L^−1^), streptomycin (50 mg·L^−1^) and kanamycin (50 mg·L^−1^) at 28 °C for 2 days. *Agrobacterium* cells were centrifuged and resuspended in infiltration solution, adjusted to an OD600 of 0.5 for infiltration into tobacco leaves with a 1 mL syringe (no needle). Before infiltration, healthy six-week-old tobacco plants were placed under a white fluorescent lamp for 1 h. After infiltration, the infiltrated plants were maintained in a controlled-environment growth chamber under normal growth conditions and identified with different tags for subsequent treatment experiments.

### 4.9. Histochemical and Fluorometric Assays for GUS Activity

For histochemical GUS staining, the infiltrated tobacco leaves were incubated in GUS staining solution with 50 mM sodium phosphate (pH 7.0), 10 mM Na_2_EDTA, 0.5 mM K_4_Fe(CN)_6_·3H_2_O, 0.1% Triton X-100 and 1 mM X-Gluc (Sigma-Aldrich, Shanghai, China) at 37 °C for 24 h and then cleared with 70% ethanol [90]. To monitor the activity of the *DkLOX3* and CaMV35S promoters, quantitative GUS assays were performed according to the previously described method [90]. The fluorescence of the methylumbelliferone products was quantified with a Hitachi 850 fluorescence spectrophotometer (Hitachi, Tokyo, Japan). The total concentration of the protein extract from the tested samples was normalized using an established protocol [91]. GUS activity was expressed as nM of 4-methylumbelliferone (4-MU, Sigma-Aldrich) generated per minute per milligram of soluble protein. The GUS measurements were repeated at least three times with similar results.

### 4.10. Statistical Analysis

Experiments were performed according to a completely randomized design. The data were tested through analysis of variance (ANOVA) using SPSS statistics 17.0, and the means were compared with the least significant difference (LSD) test. *p*-Values below 0.05 were considered statistically significant (*p* < 0.05). All measured values were presented as the mean ± standard error of the means.

## 5. Conclusions

In summary, we employed various microscopic techniques to identify qualitative changes in the fruit peel structure in two persimmon cultivars with different postharvest ripening rates. Additionally, DkLOX3 was successfully localized in the membranes surrounding the cytoplasm, plastids and mitochondria. During storage, greater amounts of DkLOX3-immunolabelled gold particles accumulated in the cell plasmalemma of “Fupingjianshi” compared with that of “Ganmaokui”. The results suggested that *DkLOX3* may play a positive role in ultrastructural changes promoting persimmon fruit ripening. In addition, we isolated the *DkLOX3* promoter, and *cis*-regulatory elements involved in the promoter sequence were analysed. Furthermore, we examined *cis*-regulatory elements to investigate the mechanisms regulating their expression under hormonal stress conditions, and two conserved hormone-responsive elements (TGACG motif and TCA-element) were found to be essential for responsiveness to hormonal stress. The results provide us with new clues for investigating the function of *DkLOX3* gene regulation in hormonal stress response.

## Figures and Tables

**Table 1:** Oligonucleotide sequences for primers used in this study.

Primer Name	Primer Sequence (5′–3′)	Purpose
pLOX3-SP1	AACTCAGTGTGGTGAAGATTGCGGATG	
pLOX3-SP2	AAGGCGACATCGAAAGCTGAATCTCC	*DkLOX3* promoter clone
pLOX3-SP3	CAAGTAAGCTGCCTTCCCAAGCTTCC	
DkLOX3-PF	*CCCAAGCTT*CCACAATTATTCTTTGGTTATTTCG	*DkLOX3* full-length promoter clone
DkLOX3-PR	*GCTCTAGA*CTTCTTCTTCTTCTTCTTCTTCTGC	
DkLOX3-PF5	*CCCAAGCTT*TATAGTATGGTCTATCATTCTCATACG	*DkLOX3* promoter deletion derivatives construct
DkLOX3-PF4	*CCCAAGCTT*GTACCCCATGTTTGATACAACTCT	
DkLOX3-PF3	*CCCAAGCTT*GCATTTCTATGTATGCTCTCTTATG	
DkLOX3-PF2	*CCCAAGCTT*CTGGATCTAGTTCAACCCATAAG	
DkLOX3-PF1	*CCCAAGCTT*TTATCAGGGATCTGATTTGTCTTA	
DkLOX3qF	CACTGCTCTTCCCTACCA	*DkLOX3* qRT-PCR
DkLOX3qR	CAGAGGGAGAAATCAGTGATACAC	
ActinqF	GGATTCTGGTGATGGTGTTAG	*Actin* qRT-PCR
ActinqR	CAGCAGTTGTTGTGAAGGAGT	

Letters “F” and “R” indicate the forward and reverse primers, respectively. Underlined sequences show restriction enzyme sites.
